# The history of venomous spider identification, venom extraction methods and antivenom production: a long journey at the Butantan Institute, São Paulo, Brazil

**DOI:** 10.1186/s40409-015-0020-0

**Published:** 2015-06-17

**Authors:** Sylvia M Lucas

**Affiliations:** Laboratório Especial de Coleções Zoológicas, Instituto Butantan, Avenida Vital Brazil, 1500, Butantã, São Paulo, SP Brazil

**Keywords:** *Ctenus*, *Lycosa*, *Phoneutria*, *Latrodectus*, *Loxosceles*

## Abstract

The article provides a historical report on venomous spider identification, venom obtainment methods and serum production at the Butantan Institute, São Paulo, Brazil. It is based on literature and personnal experience during the last 50 years. This result is the discovery that the real species causing potential severe human accidents were the spiders of the genus *Loxosceles* and *Phoneutria*.

## Background

Cases of envenomation by spider bites have been well known since ancient times. Aristotle had already identified several types of spiders: “Of spiders and phalangia there are many species. Of the venomous phalangia there are two; one that resembles the so-called wolf-spider, small, speckled, and tapering to a point […] the other kind is large, black in color, with long front legs” [1].

During the Middle Age, tarantism was well disseminated in southern Italy and its symptoms included fear sensation, chest oppression, dyspnea, intense agitation and visual disturbances, among others. At the time, these accidents were attributed to bites of a spider common in the region, referred to as “tarântula”. Later, the accidents were clarified and the responsible spiders identified as *Latrodectus* Walckenaer, 1805 (common name: black widow)*.*

In South America, there were many reports of accidents involving mostly spiders of the genera *Latrodectus* and *Loxosceles* Heineken & Lowe, 1832, which occurred in Argentina, Chile and Bolivia. These reports had descriptions of the accidents and the patients’ symptoms, but lacked information on venom studies.

## Review

In 1924, Vital Brazil and Vellard [2], while working at the Butantan Institute in São Paulo, Brazil, presented pioneering studies on venomous spiders and described the symptomatology and treatment of accidents. They made a comprehensive review of the published articles on arachnidism and concluded that knowledge on venom effects was not only imperfect but also less complete when compared to clinical studies. Furthermore, they noted that most taxonomic identification of spiders were incorrect.

For their studies, the following species were selected: *Ctenus ferus* (Perty, 1833) (= *Phoneutria nigriventer*), *C. nigriventer* Keyserling, 1891 (= *Phoneutria nigriventer*), *Nephilla cruentata* (Fabricius, 1775) (= *Nephilingis cruentata*), *Trechona venosa* (= *Trechona rufa* Vellard, 1924) and *Lycosa erythrognatha* Lucas, 1836. They believed that these spiders were the main species responsible for human accidents. Two cases that happened in 1917 were described: the first one took place at the Butantan Institute and the second at Niterói, Rio de Janeiro, while the spider was being caught with a forceps. They also described a third accident, in which a child presented a large necrotic area on the chest that was attributed to a *Lycosa erythrognatha* bite [2]*.*

Vital Brazil observed that *L. erythrognatha* was a very common spider in cold and temperate regions, and that it usually inhabited old houses and gardens, preferring dry grounds, living under stones, rotten wood and holes in ravines. One of his patients described the spider that caused his accident as being a specimen of average size and grey or dark grey in color. Later, this patient brought some specimens similar to the ones he believed had caused the accident, which were identified as *Lycosa raptoria* (= *L. erythrognatha*). Since then, almost all accidents with severe necrosis were erroneously attributed to *Lycosa* Latreille, 1804 [2]*.*

For a second study carried out in 1926, Vital Brazil and Vellard [3] extended their work by including information on the venom of some mygalomorph spiders, such as *Grammostola* Simon, 1892 and *Acanthoscurria* Ausserer, 1871*.* They observed the effects of these spiders’ venom on cold-blooded animals and concluded that the venom of *Grammostola actaeon* (Pocock, 1903) was extremely active in snakes but almost inactive in humans. More than 30 accidents were reported between April 1925 and August 1926, for which a specific antivenom was used for the first time. Based on the identification made by researchers, five of the accidents were caused by *Lycosa raptoria* and eight by *Ctenus ferus*. The causative agents of the other accidents were identified through only the symptomatology and patient description of the spider. Two patients developed widespread necrosis, and a serum against both *Ctenus* Walckenaer, 1805 and *Lycosa* spider venom was used for treatment (*Ctenus*-*Lycosa* antivenom).

Later, in 1936, Vellard [4] continued his study by including information on several species of araneomorph and mygalomorph spiders. He described the venom apparatus of the species, the histology of venom glands and their biting mechanism. Additionally, he estimated the quantity of injected venom by comparing the weights of glands extracted full of venom, both before and after squeezing. Studies on physicochemical properties of venoms and their action on different laboratory animals were presented. Curiously, he considered the venom of *Loxosceles* spiders to be of low potency in human beings. Thus, for many years, the Butantan Institute did not worry about the production of a specific antivenom against these spiders, although accidents with severe symptomatology had been reported in other South American countries.

In 1952, Bücherl [5] also working at the Butantan Intitute, continued studying spider venom and improved the methodology of obtaining pure venom by using electrical stimulation of the spiders or by dissecting their venom glands. He described the venom effect on several laboratory animals and concluded that the ones extracted from large tarantulas, such as *Grammostola* sp.*,* did not act on humans. Among araneomorph spiders, he distinguished two different types of venom effects. The first caused superficial skin necrosis, papules, vesicles, edema, urticaria, general weakness, diarrhea and hemoglobinuria. As causative agents of these symptoms, he included the venom of the following spiders: *Lycosa, Nephila, Loxosceles* and the less active *Argiope* Audouin, 1826, *Neoscona* Simon, 1864 and *Filistata* Latreille, 1810. The second group included spiders whose venoms acted on the nervous or renal system, as *Phoneutria*, *Ctenus* and *Latrodectus* (and perhaps *Enoploctenus* Simon, 1897).

One year later, in 1953, Bücherl [6] developed a method to obtain pure venom from *Phoneutria* species, using two long glass pipettes joined by a thin elastic rubber tube. This device was introduced into the cage of the spider and, when bitten by the animal, drops of venom were collected. The process was repeated with different specimens until enough venom was obtained. Then, the rubber tube was disconnected and the liquid venom blown over a clear glass and dried in a vacuum desiccator. This process resulted into pure dry venom that could be stored.

In the same year, Bücherl [7] began the use of electric stimulation for obtainment of *Phoneutria* venom. This methodology had already been tested by Barrio and Vital Brazil in 1949 [8], with mygalomorph spiders and has since been improved, enabling the use of venoms in important research studies.

With regard to the genus *Loxosceles*, Bücherl [9] studied the venom of two different species, which he identified as *L. laeta* (Nicolet, 1849) and *L. rufipes* (misidentified as *L. gaucho* Gertsch, 1967). At first, he used venom glands extracted from living animals and tested them on laboratory animals. He concluded that: “due to the small size of the spiders and their small fangs unable to introduce the venom in deeper layers of the skin, these species will never be fatal to humans*”*. Moreover, he observed that *Loxosceles* are spiders commonly found inside houses and that their symptomatology is very similar to that of *Lycosa*, making it very difficult to distinguish between accidents caused by spiders of the two genera. As treatment for accidents with these species, he recommended the use of an antivenom against *Ctenus* and *Lycosa* spider venom (*Ctenus-Lycosa* antivenom). The admission of several patients with deep and severe skin necrosis caused by spider accidents was registered by physicians at the Vital Brazil Hospital, at the Butantan Institute, for several years. However, as the patients did not bring the spider that caused the bite, the species could not be identified in most of the cases. Only when technicians of the institute searched for spiders at the location of the accidents and found specimens of *Loxosceles*, could “loxoscelism” be confirmed.

In 1961, an experimental antivenom for *Loxoceles* was produced, when venom, from poison glands preserved in glycerin, was used to immunize two horses. This serum was employed for the first time in a bitten patient that went to a hospital 60 h after the accident and, besides extensive necrosis, presented with other severe symptoms including kidney problems. The experimental serum was applied only six days after the accident and the patient recovered fifteen days later, although he needed plastic surgery [10]. After the experimental production of *Loxosceles* antivenom production started in 1964.

Since 1961, occurrence of black widow spiders was observed along beaches in the state of Rio de Janeiro and the former state of Guanabara, due to which three accidents were reported. Bücherl [11] identified the species as belonging to the *Latrodectus curacaviensi*s group. Numerous living specimens were collected and the Butantan Institute intended to produce an antivenom against *Latrodectus* spiders. However, the work was interrupted due to several factors: the transport of the living spiders to the institute, the venom extraction from very few specimens and the low number of accidents. Nowadays the Vital Brazil Institute in Niterói, Rio de Janeiro, Brazil, is responsible for a regular production of *Latrodectus* antivenom.

Since the first studies on spider venoms at the Butantan Institute, researchers have had to overcome many difficulties including: linking the accidents to the spider and performing a correct taxonomic identification, collecting enough spiders to obtain the necessary venom, and improving the methodology for obtainment of high quality venom. It has been, therefore, a long journey…

## Conclusion

The improvement of different methods to get specimens, better animal care conditions in captivity and venom extraction techniques offered the opportunity to study the arachnid venoms under different perspectives and better understand how they act on humans [12].

The flowing list shows, in chronological order, the spider antivenoms produced at the Butantan Institute (personal communication of Dr. Ivone K. Yamaguschi, responsible for antivenom production at the institution):

- 1924–1925: *Ctenus* antivenom and *Lycosa* antivenom produced on small scale from 1926 onwards.
- September 26, 1958: *Lycosa* antivenom and *Ctenus* antivenom.
- August 9, 1960*:Lycosa* antivenom and *Ctenu*s antivenom.
- June 18, 1963: *Ctenus-Lycosa* antivenom.
- July 21, 1964: *Ctenus* antivenom, *Lycosa* antivenom and *Loxosceles* antivenom (produced until 1984).
- December 23, 1984: *Loxosceles* antivenom and *Phoneutria* antivenom.
